# Unveiling disulfidptosis-related genes in HBV-associated hepatocellular carcinoma: an integrated study incorporating transcriptome and Mendelian randomization analyses

**DOI:** 10.7150/jca.93194

**Published:** 2024-08-26

**Authors:** Xilong Wang, Ke Xiao, Zhipu Liu, Li Wang, Zhaogang Dong, Hongxing Wang, Yuhui Wang

**Affiliations:** 1 Department of Clinical Laboratory, Weifang People's Hospital, Weifang, 261000, China.; 2 Department of Clinical Laboratory, Qilu Hospital of Shandong University, Jinan, 250012, China.; 3 Shandong Engineering Research Center of Biomarker and Artificial Intelligence Application, Jinan, 250012, China.; 4 Department of Clinical Laboratory, The First Affiliated Hospital of Anhui Medical University, Hefei, 230032, China.

**Keywords:** Disulfidptosis, HBV-HCC, Mendelian randomization, Prognosis, GYS1

## Abstract

Disulfidptosis, a recently unveiled mechanism of demise, has been linked to an unfavorable prognosis in the context of hepatocellular carcinoma (HCC). However, few studies have focused on the causal link between disulfidptosis and HBV-related HCC (HBV-HCC). In this study, the Mendelian randomization (MR) analysis demonstrated that the risk of HCC increased with increasing genetic susceptibility to HBV, and the genetic changes of disulfidptosis were significantly associated with the increased risk of HBV-HCC. Within both the TCGA and GEO cohorts, it is possible to accurately forecast the prognosis of HBV-HCC by utilizing a risk score that is derived from a combination of GYS1, RPN1, SLC7A11, LRPPRC and CAPZB genes. GYS1, a potential therapeutic target for HBV-HCC, exhibits a remarkable positive correlation with immune infiltration and MSI when compared to other molecules. Furthermore, we demonstrated that silencing GYS1 effectively inhibits the tumor proliferation and metastasis of HBV-HCC *in vitro and in vivo*. Overall, this study expands the understanding of the potential roles of disulfidptosis in HBV-HCC and highlights GYS1 as a promising target for HBV-HCC.

## Introduction

Hepatocellular carcinoma (HCC), an extremely prevalent malignancy, ranks as the third highest contributor to cancer-related mortality on a global scale 1. Chronic infection with the hepatitis B virus (HBV) can instigate both acute and chronic liver ailments, serving as a primary predisposing factor in the progression of HCC 2. Based on statistical data, it is estimated that approximately 350 million individuals worldwide suffer from persistent HBV infection, resulting in an alarming annual mortality rate of over 600,000 patients due to complications associated with chronic HBV infection 3. In fact, in China, over 80% of HCC cases are associated with chronic HBV infection, commonly known as HBV-associated HCC (HBV-HCC) 4,5. Despite the continuous advancements in the treatment of HBV-HCC, such as surgical resection, liver transplantation, radiotherapy, molecular targeted therapy and antiviral therapy, the long-term survival rate of HBV-HCC patients remains disappointingly low 6,7. Hence, it is crucial to determine supplementary biomarkers that are both heightened in sensitivity and specificity concerning the prognosis of HBV-HCC. This is indispensable in foreseeing the prognostication of patients afflicted with HBV-HCC and constructing personalized approaches to treatment.

Disulfidptosis, a newly identified form of cell demise triggered by disulfide stress, was investigated in a study conducted by Liu *et al.* The research findings revealed that cells deprived of glucose and overexpressing SLC7A11 exhibited a substantial depletion of intracellular reduced nicotinamide adenine dinucleotide phosphate (NADPH) while experiencing abnormal accumulation of cystine 8. Gradual and substantial buildup of disulfides, such as cystine, then initiates the formation of actin cytoskeletal disulfide bonds and subsequent cytoskeletal contraction, eventually culminating in disulfide stress and swift cellular demise 9. Chen *et al.* have previously developed a risk model that utilizes immune checkpoint-related genes and DRGs to forecast the prognosis of HCC 10. Nevertheless, the prognostic value of DRGs in HBV-HCC has not been studied and confirmed.

In this study, Mendelian randomization (MR) analysis was first conducted to investigate the potential causality between disulfidptosis and HBV-HCC risk. By utilizing univariate Cox regression and LASSO regression analysis, we constructed a 5-gene signature. Moreover, we also explored the association between signature of disulfidptosis and immune infiltration into tumors, tumor mutation burden (TMB), microsatellite instability (MSI), and chemotherapeutic drug responsiveness. Ultimately, we assessed the impact of GYS1, a prognostic molecule, on the growth and metastasis of HBV-HCC tumor both* in vitro and in vivo*. The study offers novel findings into the prediction of risk levels and the use of immunotherapy in individuals with HBV-HCC. Furthermore, the exploration of GYS1 as a potential therapeutic target may present itself as a feasible approach in the management of HBV-HCC.

## Materials and methods

### Mendelian randomization analysis

Data and participants were obtained from previous Genome-wide Association Studies (GWAS) focusing on HBV-HCC, including a total of 1,538 patients with HBV-positive HCC and 1,465 HBV-positive individuals serving as controls. All participants were Han Chinese from East and South China 11. SNPs strongly associated with HBV or disulfidptosis were screened for further analysis. A series of quality control steps were performed based on the GWAS results to select qualified instrumental variables. Initially, we employed an inverse variance weighted (IVW) meta-analysis approach to evaluate the causal link between exposure and outcome by analyzing each Wald ratio. Additionally, we employed a "leave-one-out" sensitivity analysis to ascertain if the observed causal effects were primarily influenced by a single potentially influential SNP. Significance of the association between exposure and outcome phenotype was determined at a P-value of less than 0.05. All MR analyses were conducted utilizing the "TwoSampleMR" R package.

### Datasets and preprocessing

Data of 145 cases of HBV-HCC tissue samples and 50 adjacent cancer tissue samples, including mRNA expression data and clinical information, were gathered from TCGA database (https://portal.gdc.cancer.gov/). Normalization of the gene expression profiles was performed using the scaling technique from the R package "limma". In addition, we obtained microarray expression profiles and clinical data for 212 cases with HBV-HCC from the GEO database (https://www.ncbi.nlm.nih.gov/geo/). Before any additional analysis, log2 (data+1) transformation was applied to all mRNA expression data. This study only included HCC patients with complete clinical data and a positive HBV infection. Patients with other viral hepatitis, autoimmune liver disease, alcoholic liver disease, and secondary liver cancer were excluded 12. The detailed clinical characteristics of these patients are summarized in Table S1. Finally, genes associated with disulfidptosis were obtained from literature that has been published before 9,13-15.

### Identification of differentially expressed DRGs

From the expression data of the TCGA cohort, we obtained the expression profiles of 23 DRGs. In order to identify these differentially expressed DRGs, we utilized the "limma" R package and generated boxplots with the assistance of the "ggplot2" R package. By applying cutoff criteria of false discovery rate (FDR) <0.05, we successfully obtained the desired differentially expressed DRGs. To construct a correlation network of the DRGs, we employed the "circlize" R package.

### Consensus Clustering

The consensus clustering algorithm, implemented with the R package "ConsensuClusterPlus", determined the quantity of clusters 16,17. The correlation between patient groups and overall survival (OS) was analyzed employing the "survival" package and the Chi-square test. The Kaplan-Meier curve can be generated using the R package "pheatmap".

### Identification of prognostic gene signature

The training set comprised of 145 HBV-HCC samples obtained from the TCGA cohort. For analysis of the training set DRGs, the "survival" package in R 4.1.2 was utilized to conduct univariate Cox regression. Survival curves for genes displaying p<0.05 were graphed. To ensure gene count reduction and minimize collinearity among genes, LASSO Cox regression was employed.

### Construction and validation of a prognostic model

Using these findings from LASSO Cox regression, we computed risk scores for every sample in the TCGA chort 18. The evaluation of disparities in OS and the creation of Kaplan-Meier survival graphs were conducted utilizing the "survival" package. Moreover, the model's predictive capability was assessed by creating a ROC curve using the "timeROC" package. Based on the identical genes and coefficients identified in the training set, we computed the risk scores for individuals in the GEO cohort, subsequently assigning them to either high or low risk groups.

### Establishment of nomogram

Univariate and multivariate cox regression analysis were used to determine the appropriate items to establish nomogram through "forestplot" package. The nomogram chart provides an illustration of a patient's risk of recurrence via "rms" package.

### Tumor immune microenvironment analysis

The "cibersort" package was utilized to perform calculations on immune cell infiltration in tumors 19. Estimations of 24 immune cell populations' abundance were carried out using the ssGSEA algorithm 20. Following this, we employed the TIMER algorithm to establish the correlation between immune cells and either a risk score or signature gene 21.

### TMB and MSI analysis

Initially, we acquired the genetic alteration information from the TCGA cohort. Afterwards, we employed the R package called "maftools" to examine the disparities in mutation status among two risk subcategories. The assessment of the correlation between the measures of 5 DRGs and TMB and MSI was conducted using Spearman correlation.

### Immune checkpoint and drug sensitivity analysis

A "ggplot2" package was employed to generate box plots showcasing the immune checkpoint expression in patients with HBV-HCC. To determine whether there is a correlation between gene expression and drug sensitivity in cancer, we performed a correlation analysis. For this analysis, we utilized two databases: GDSC database (https://www.cancerrxgene.org) and CTRP database (https://portals.broadinstitute.org/ctrp/). These databases were selected as they provide comprehensive information on gene expression and drug sensitivity in cancer.

### Cell culture and transfection

Human hepatoma cell lines PLC/PRF/5 and SNU182 were purchased from the Cell Bank of the Chinese Academy of Sciences (Shanghai, China). The cells were cultured in DMEM supplemented with 10% FBS. The incubator maintained an environment of 37°C and 5% CO_2_. GYS1-shRNA plasmid (sequence: 5′-AATTCCGCTATGAGTTCTCCAACAA CTCGAG TTGTTGGAGAACTCATAGCGG-3′) and negative control shRNA (sequence: 5′-AATTCCTAAGGTTAAGTCGCCCTCGCTCGAGCGAGGGCGACTTAACCTTAGG-3′) were purchased from GeneChem (Shanghai, China). After 24 hours of lentiviral infection, stable integrants were chosen using puromycin. The level of GYS1 silencing achieved by shRNA was then evaluated employing qRT-PCR.

### qRT-PCR

The TRIzol kit (Invitrogen) was employed in extracting RNA from cells. The Prime Script RT reagent kit (Vazyme) was then utilized to reverse transcribe the isolated mRNA. Conducting qRT-PCR, the SYBR Green mix (Vazyme) was used with GAPDH as the reference gene. The analysis of data followed the 2-ΔΔCt method. The primer pairs used for GYS1 amplification were as follows: forward, 5′-CAGACAGTGGTTGCCTTCTTC-3′; reverse, 5′-TTCCTCCCGAACTTTTCCTT-3′.

### Cell proliferation assay

In order to perform the CCK-8 test, we readied the cells by placing them in 96-well plates at a density of 1 × 10^3^ cells per well. We introduced 10 μl of CCK-8 solution (Dojindo) into each well at regular time intervals, then incubated it at 37°C for 2 hours. To determine cell viability, we measured the absorbance at 450 nm. For the colony formation experiments, we took 500 transfected cells and seeded them in each well of a 6-well plate. Afterward, these cells were cultured in DMEM enriched with 10% FBS for about two weeks. After the period of incubation, we treated the colonies with methanol, applied a 0.5% crystal violet solution for staining, and ultimately observed and tallied them.

### Cell invasion and migration assay

Cell migration and invasion capacities were assessed using a modified two-well culture system with an 8 μm pore size. The invasion or migration experiments were conducted utilizing transwell chambers (Corning), with or without Matrigel (BD Biosciences) coating, respectively. To the upper chamber, a 150 μl suspension of DMEM lacking fetal calf serum (3×10^4^ cells/well) was introduced, whereas to the lower chamber, a 600 μl suspension of DMEM with 10% fetal calf serum was introduced. After being incubated at a temperature of 37 °C for a duration of 48 hours or longer, the cells were immobilized using a solution of 4% paraformaldehyde and then subjected to staining with 0.1% crystal violet. Afterwards, the EVOS M5000 microscope (Thermo Fisher Scientific) was utilized to capture and record images, and the number of cells that had entered the wells was measured using ImageJ software.

### Nude mouse xenograft model

Male BALB/c nude mice, aged six weeks, were acquired from Jinan Pengyue Experimental Animal Breeding Co., Ltd located in Shandong, China. The mice were kept in a facility for animals that had rigorous measures in place to control the spread of pathogens. They were housed in an environment with alternating 12-hour periods of light and darkness, and they were provided with unrestricted availability of food and water. To assess the tumorigenicity, subcutaneous injection of PLC/PRF/5 cells (4×10^6^) was performed in every nude mouse. A modified version of the ellipsoidal formula was employed to compute the tumor volume, denoted as TV (mm^3^) = (L × W^2^) / 2, where L represents the long diameter and W represents the wide diameter. Following the sacrifice of the mice, the tumors were removed and their weight was measured.

### Statistical analysis

Data analysis was conducted using the GraphPad Prism 9.4.1 software. The statistical significance of the results was assessed through the utilization of Student's t-test and Two-way ANOVA analysis. Significance was determined at a p-value below 0.05.

## Results

### The causality of disulfidptosis on HBV-HCC risk

**Figure 1:**
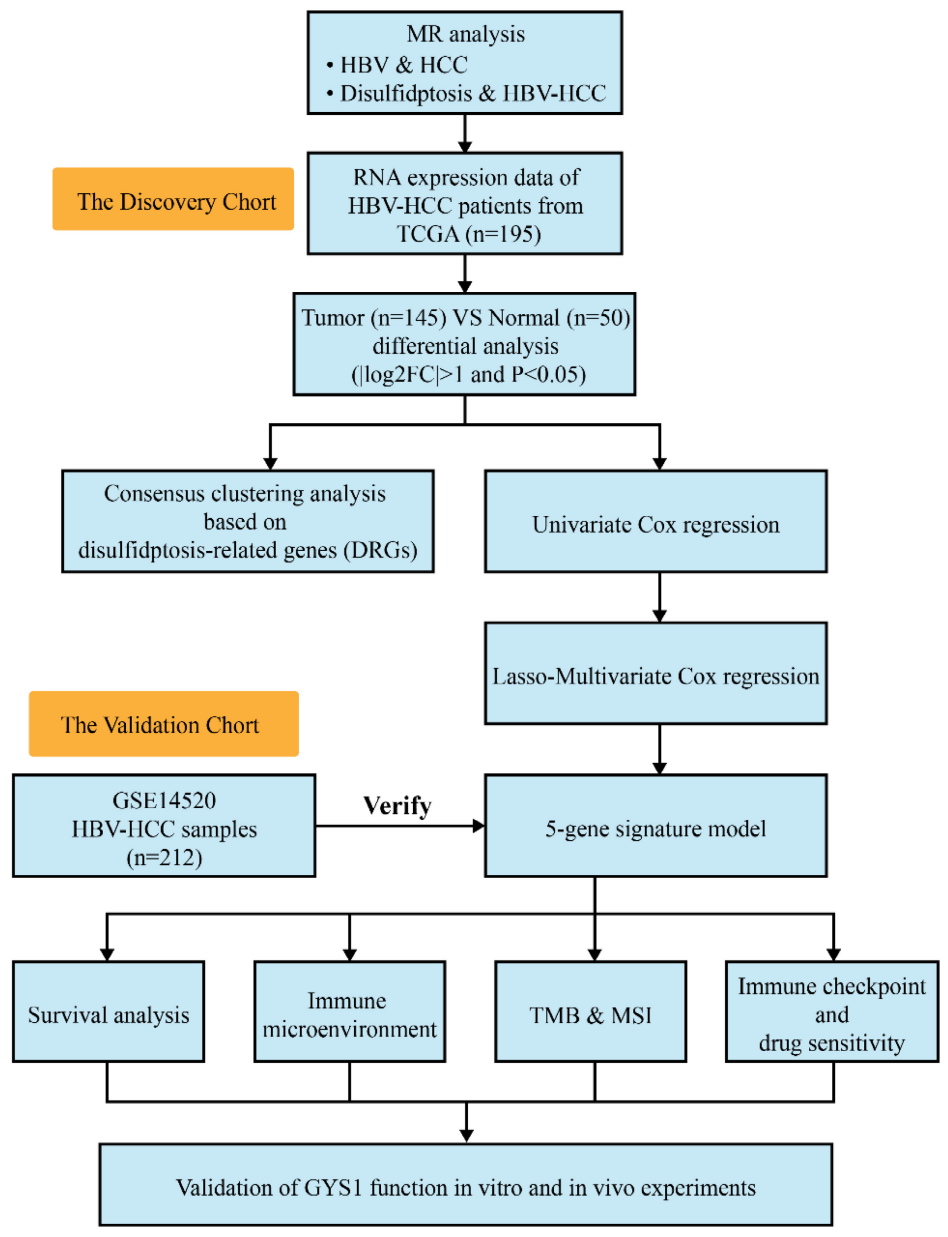
The flow chart of this study.

**Figure 2:**
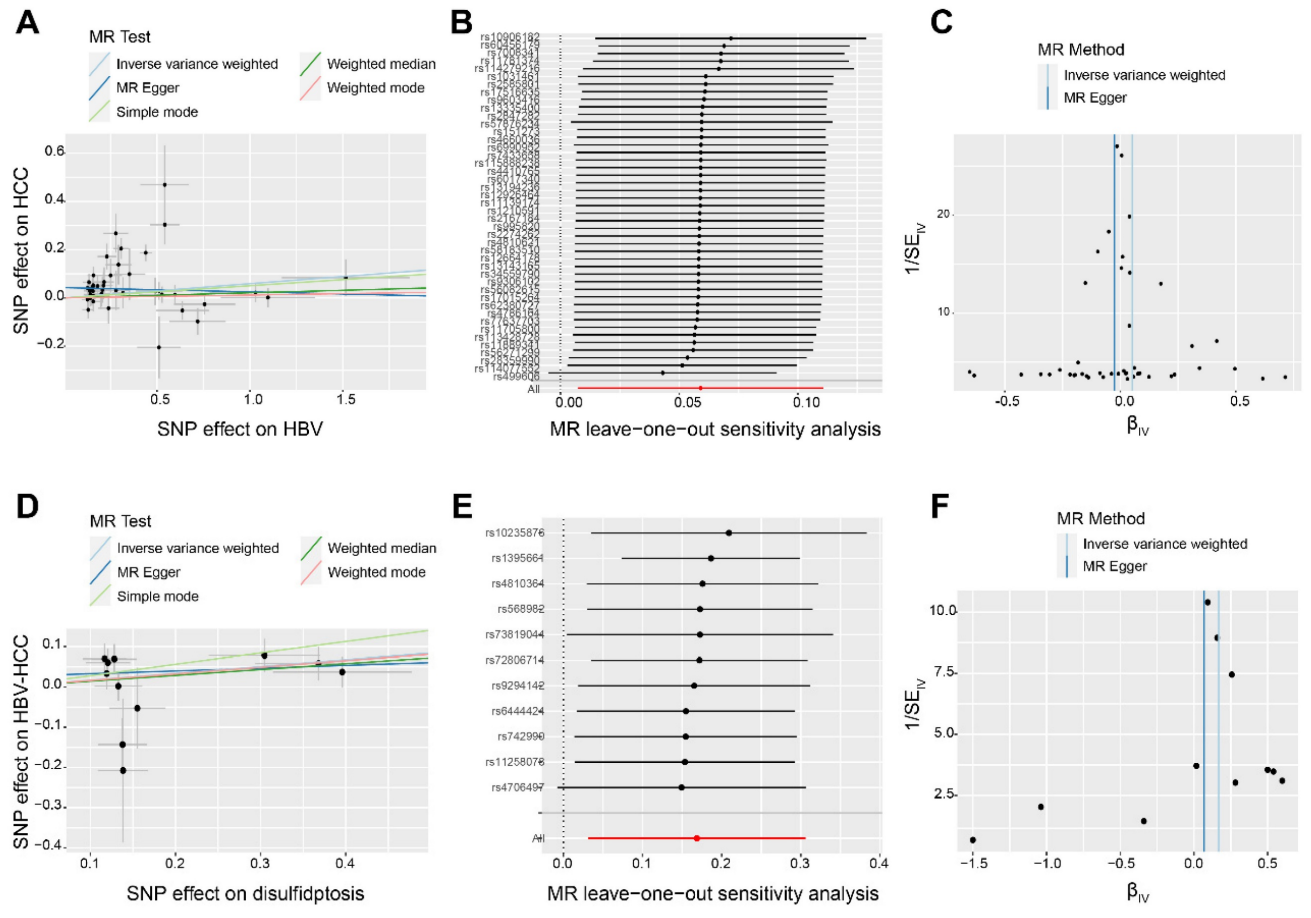
The causality of disulfidptosis on HBV-HCC risk. Scatter plot (A), leave-one-out sensitivity analysis (B), and funnel plot (C) of the effect of HBV on HCC. Scatter plot (D), leave-one-out sensitivity analysis E, and funnel plot F of the effect of disulfidptosis on HBV-HCC. The slope of each line in the scatter plot indicates the estimated effect of each mendelian randomization method. In leave-one-out analyses, black points depict the IVW method used to assess causal effects, and red points depict the inverse variance weighted estimates using all SNPs. Vertical lines indicate the estimates of all SNPs in the funnel plot.

Figure 1 presents the depiction of the flow of the study. First, we have chosen 85 SNPs as our instrumental variables in order to examine the causal connection between HBV and HCC. Analysis of IVW revealed a robust positive association between elevated genetic susceptibility to HBV infection and an increased risk of HCC (OR 1.18; 95%CI 1.007-1.117; P=0.016) (Figure 2A). In the leave-one-out analysis of HBV correlations with HCC, no putative outlier SNPs were identified (Figure 2B). The absence of any violations of the MR assumptions was suggested by the observed symmetry in the funnel plots (Figure 2C). Furthermore, we employed 14 SNPs as instrumental variables to examine the causal links between disulfidptosis and HBV-HCC. While ensuring the stability of the data, we uncovered compelling evidence indicating that genetically predicted disulfidptosis had a positive causal inference on HBV-HCC (OR 1.06; 95%CI 1.032-1.358; P=0.025) (Figure 2D-F). This implies that disulfidptosis poses as a potential hazard for HBV-HCC.

### Detection and enrichment analysis of differentially expressed DRGs in HBV-HCC

**Figure 3:**
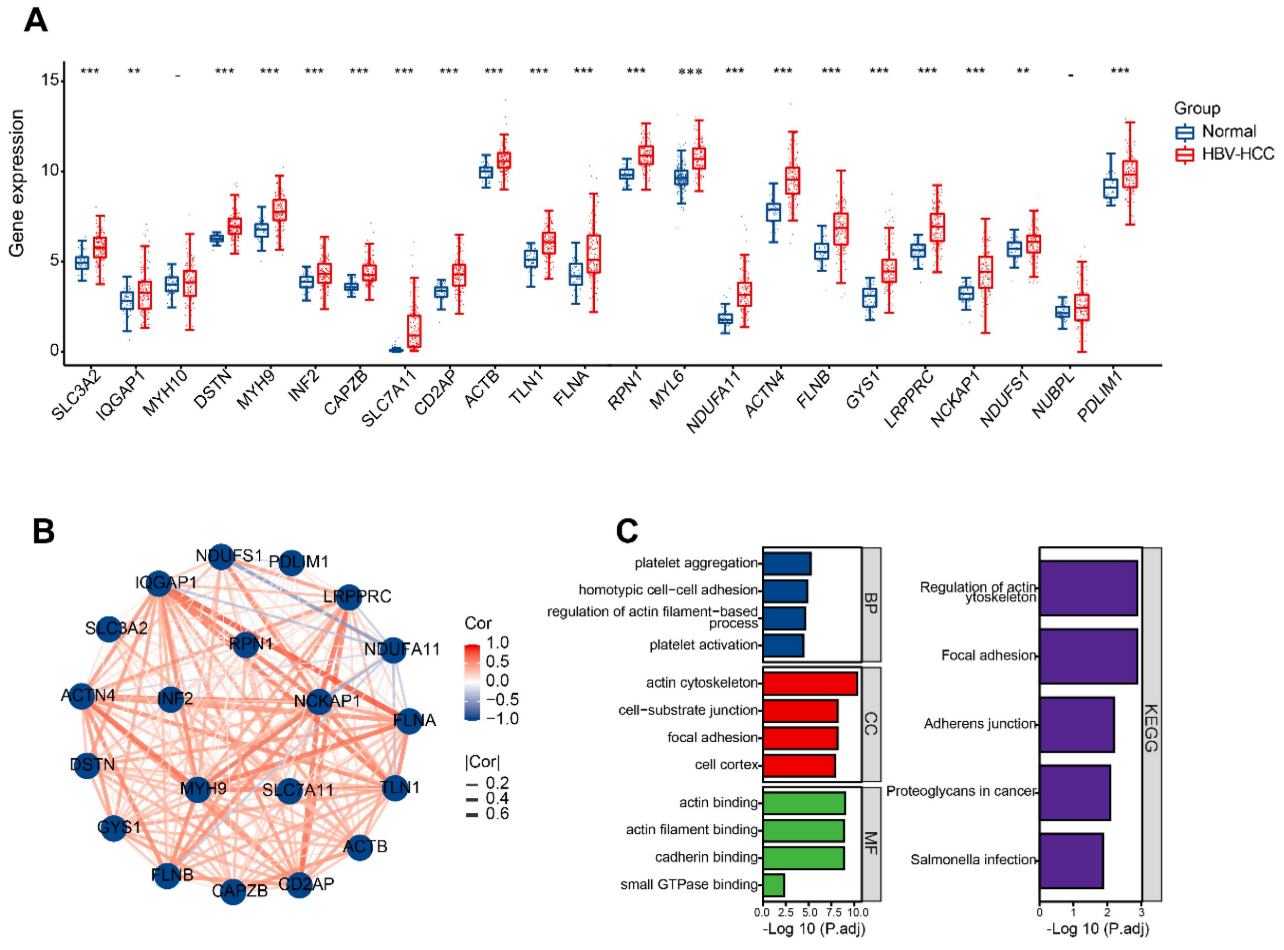
Identification and functional enrichment of differentially expressed DRGs. (A) The boxplot presented the differentially expressed DRGs comparing normal tissues in HBV-HCC. (B) The correlation network of differentially expressed DRGs. Red represents positive correlations while blue represents negative correlations. (C) GO and KEGG enrichment analysis of DRGs. *P < 0.05, **P < 0.01, and ***P < 0.001.

Initially, it was discovered that 21 DRGs exhibited a significant increase in expression in HBV-HCC tissues when compared to normal tissues (Figure 3A). Figure 3B shows the interaction network between these DRGs in HBV-HCC. The analysis of GO enrichment revealed that DRGs were mainly enriched in actin cytoskeleton, focal adhesion, platelet aggregation, homotypic cell-cell adhesion, actin binding and cadherin binding. According to the KEGG enrichment analysis, the DRGs were mainly enriched in regulation of actin cytoskeleton (Figure 3C).

### HBV-HCC classifification based on DRGs

**Figure 4:**
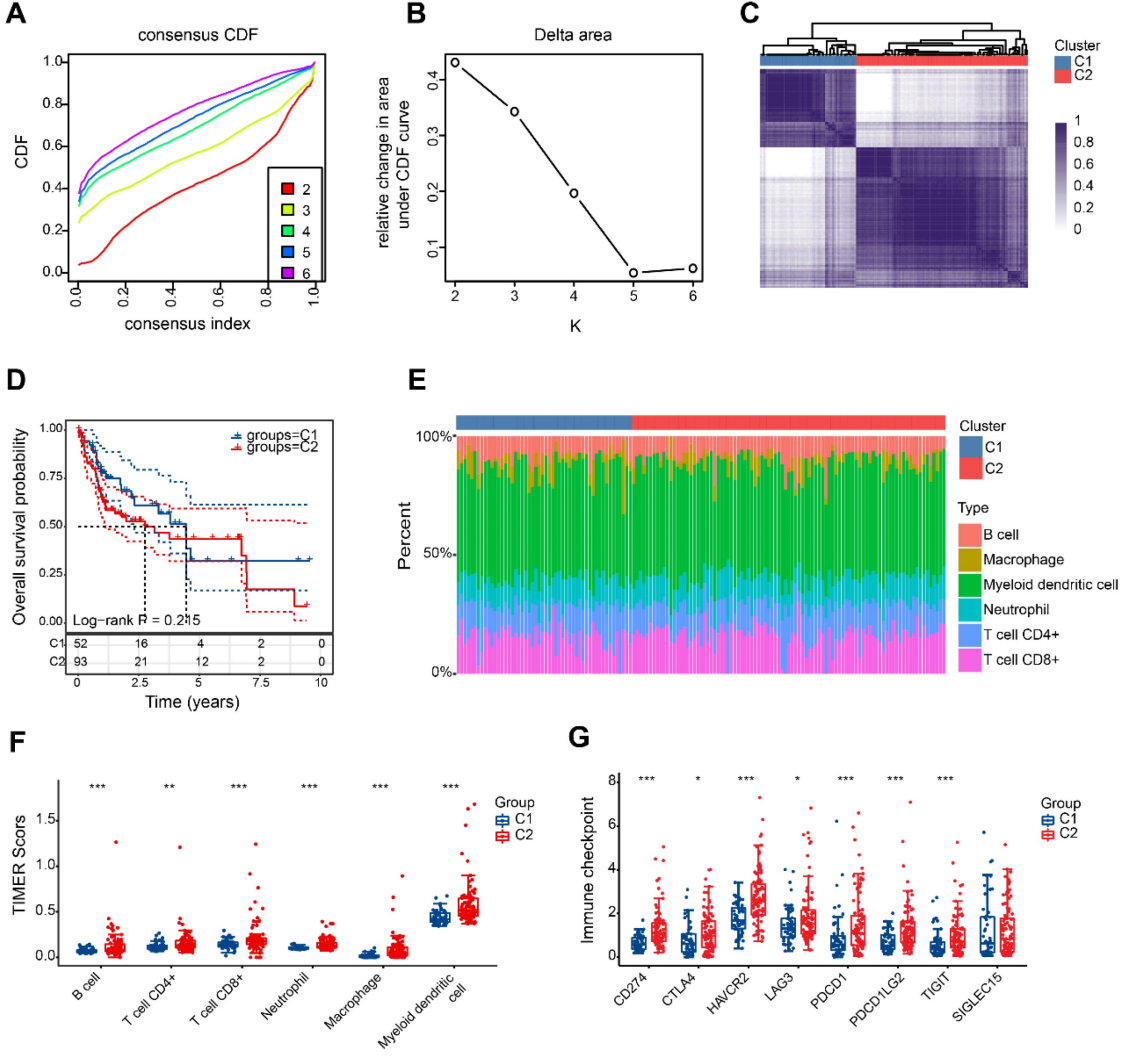
HBV-HCC classification based on DRGs. (A) The cumulative distribution function (CDF) curves from k = 2 to 6. (B) Delta area of CDF curves. (C) Consensus matrix heatmap defining two clusters (k = 2). (D) Kaplan-Meier curve of the prognostic relationship between the two clusters in the HBV-HCC cohort. (E) The percentage of immune infiltrating cells in two subgroups using TIMER databases. (F) Boxplots depicting the immune infiltration levels within two subgroups. (G) Boxplots depicting the immune checkpoint expression within two subgroups. *P < 0.05, **P < 0.01, and ***P < 0.001.

According to the findings, a value of 2 for k is the optimal choice for achieving stable clustering (Figure 4A, B). As shown in Figure 4C, HBV-HCC samples were divided into 2 clusters, namedly, C1 (n=52) and C2 (n=93). The two groups of patients showed no significant variation in overall survival (Figure 4D). Significant variations in the levels of immune cell infiltration were observed between the two groups. Figure 4E and F show that patients in the second category exhibited increased immune infiltration. It is intriguing to observe that there was also a significant upregulation in the expression of immune checkpoints within group 2 (Figure 4G).

### Establishment and validation of the DRGs prognostic model

**Figure 5:**
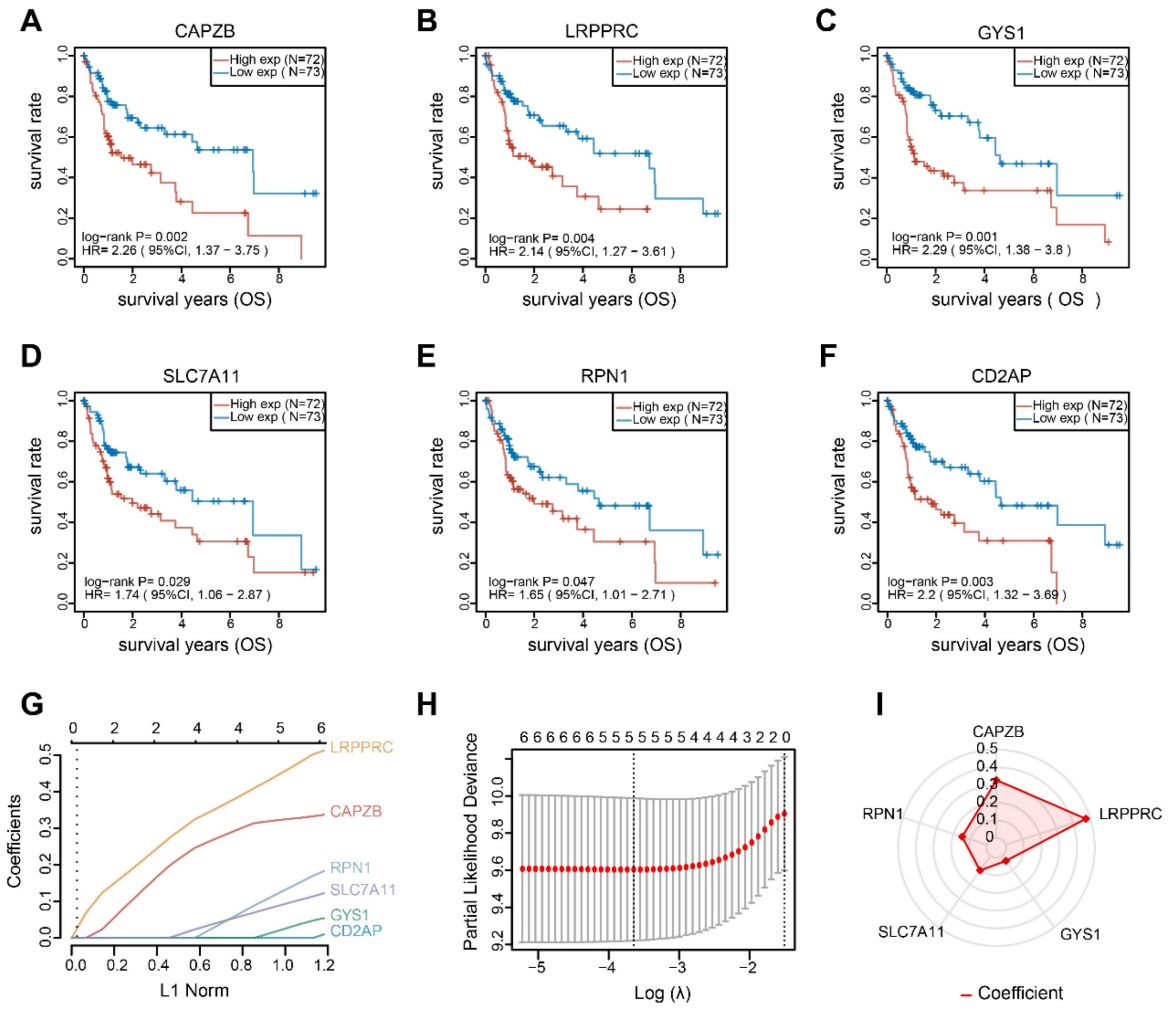
Identifying prognostic-related DRGs. The overall survival curves of CAPZB (A) LRPPRC (B) GYS1 (C) SLC7A11 (D) RPN1 (E) and CD2AP (F) in HBV-HCC patients in the high- and low-expression groups. (G) LASSO coefficient profiles of the 6 survival-related DRGs. (H) Ten-fold cross-validated LASSON regression was used to identify 5 prognostic genes with minimum Lambda. (I) Value of each coefficient representing its relative contribution to the predictive signature.

By conducting univariate Cox regression analysis, we discovered that 6 out of the total 21 DRGs exhibited a significant correlation with OS in patients with HBV-HCC. The Kaplan-Meier curves indicated that individuals with elevated expression of these DRGs exhibited unfavorable prognosis (Figure 5A-F). Afterwards, we conducted LASSO Cox regression analysis on these notable DRGs and identified 5 genes (RPN1, SLC7A11, GYS1, LRPPRC and CAPZB) that are associated with prognosis. These genes were used to develop a prognostic model (Figure 5G, H). Figure 4I shows the regression coefficient corresponding to each DRGs. The risk score = (0.3279) × CAPZB + (0.4764) × LRPPRC + (0.0356) × GYS1 + (0.1024) × SLC7A11 + (0.1483) × RPN1. Patients with HBV-HCC were separated into two groups based on the risk score.

**Figure 6:**
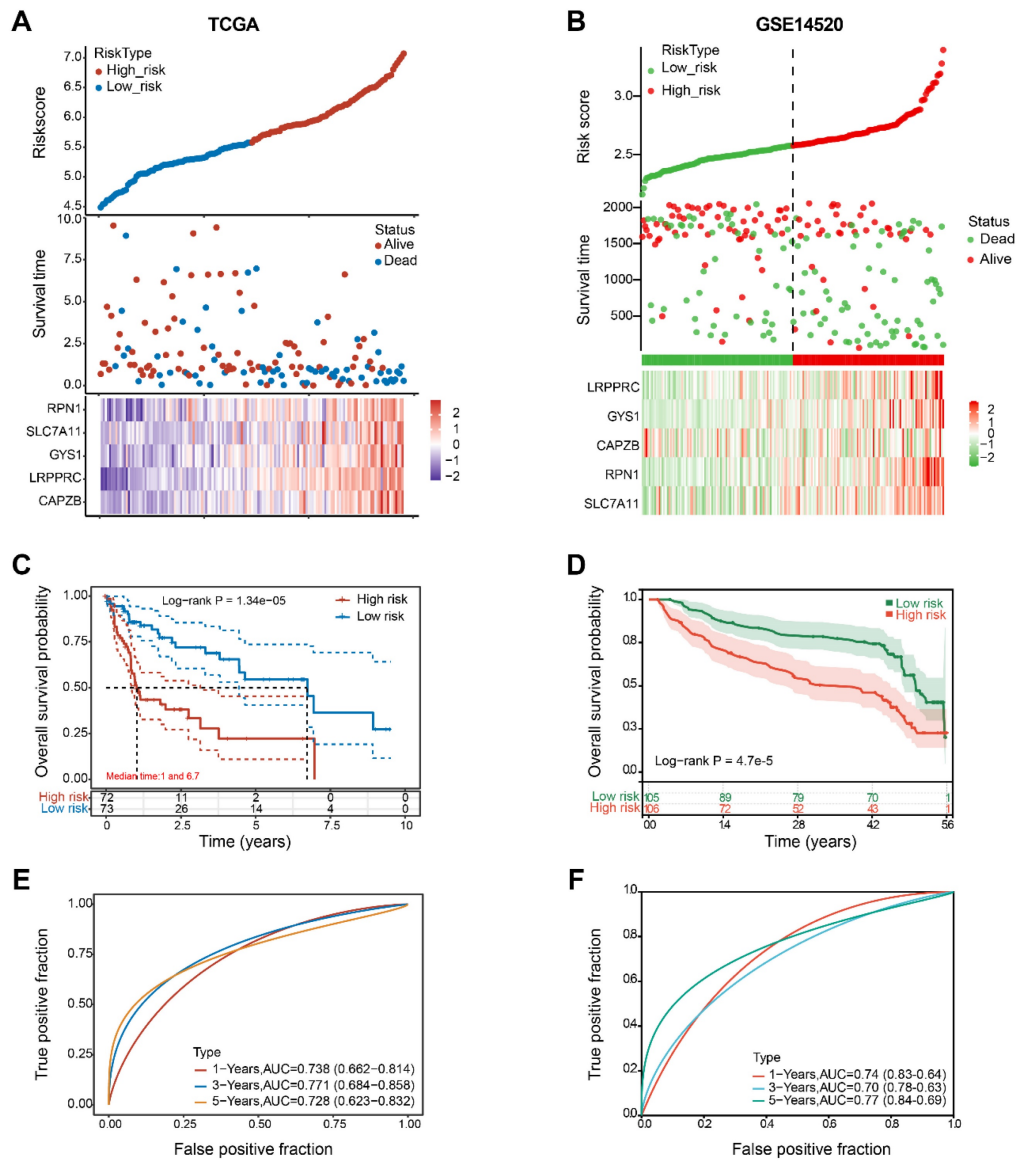
Construction and validation of 5-gene signature model. (A, B) Distribution of risk scores, cumulative mortality rate, and expression of five DRGs among HBV-HCC in the TCGA and GSE14520 cohorts. (C, D) Kaplan-Meier survival curves for the OS between the H-group and L-group in the TCGA and GSE14520 cohorts. (E, F) AUC of time-dependent ROC curves verified the prognostic performance of the risk score between the H-group and L-group in the TCGA and GSE14520 cohorts.

Figure 6A revealed that as the risk score increases, the risk of death among patients in the TCGA cohort also increases. As indicated by the Kaplan-Meier curve, individuals in the H-group had a lower overall survival rate compared to those in the L-group (with median survival times of 1 and 6.7 years, respectively, Log rank P = 0.0000134) (Figure 6C). The 5-gene signature was found to have a significant prognostic value in determining the survival rates of patients with HBV-HCC. The ROC curves demonstrated this, with an area under the curve (AUC) of 0.738 at 1 year, 0.771 at 3 years, and 0.728 at 5 years (Figure 6E).

The GSE14520 cohort was utilized to validate the prognostic value of the molecular signature on HBV-HCC. Using the identical calculation formula as the TCGA cohort, the HBV-HCC patients in this chort were divided into H-group and L-group. A correlation was noted between the escalating risk score and the progressive rise in cumulative mortality rate among HBV-HCC patients (Figure 6B). Specifically, individuals classified as high-risk experienced an increased probability of premature mortality and a reduced life span (Figure 6D). Additionally, the AUC values for the 5-gene signature were 0.74 at 1 year, 0.70 at 3 years, and 0.77 at 5 years (Figure 6F).

### Construction of a predictive nomogram

**Figure 7:**
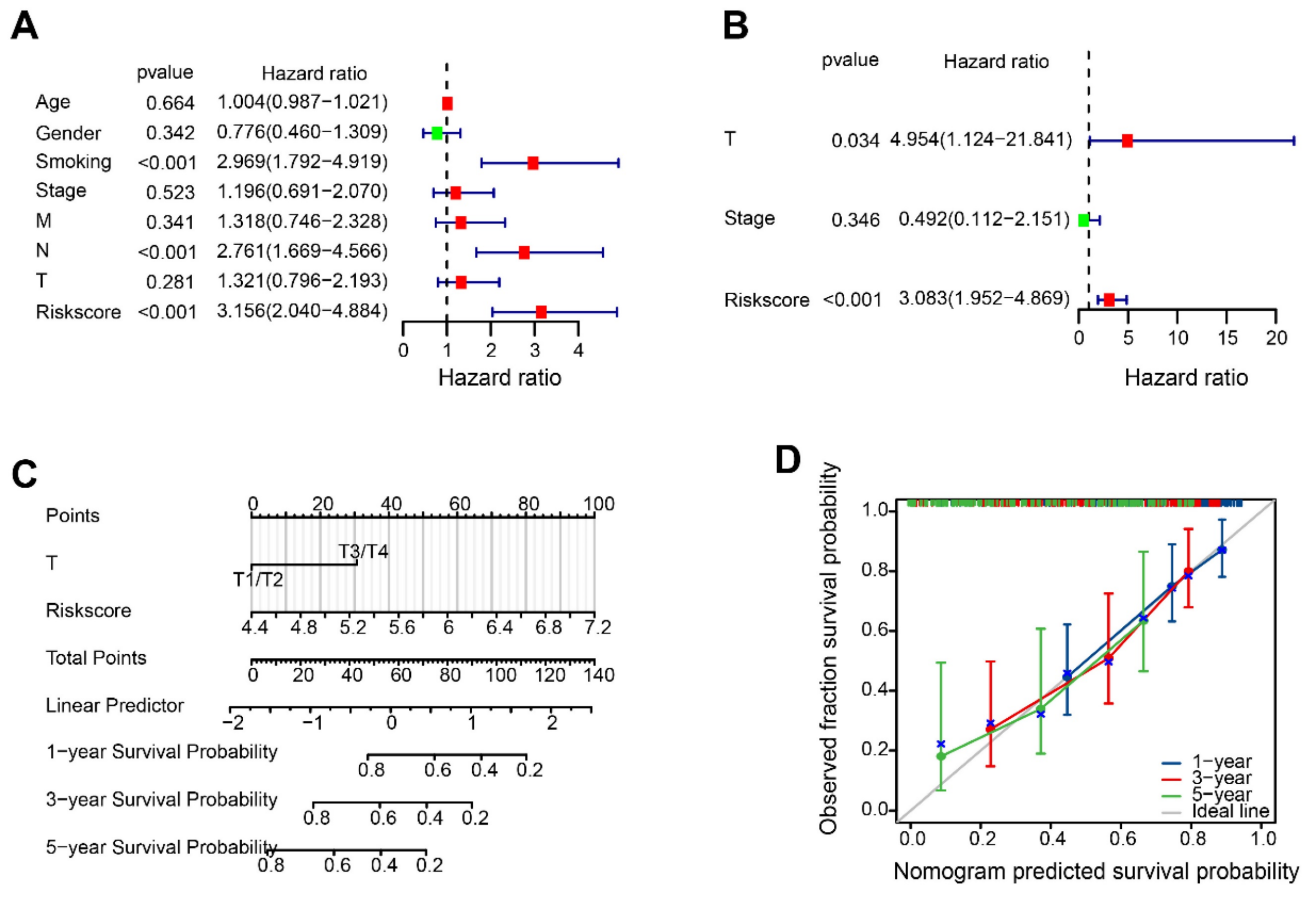
The disulfidptosis-related nomogram. (A, B) Univariate and multivariate Cox regression analysis of risk score and clinical characteristics. (C) Nomogram for forecasting OS with independent prognostic factors of HBV-HCC. (D) Calibration curves showing the accuracy of nomogram predicting the 1-year, 3-year, and 5-year OS.

According to the results of the Univariate Cox analysis, T-stage, stage and risk score were identified as significant prognostic factors with a high-risk association (Figure 7A). The prognosis of HBV-HCC was influenced independently by T-stage and risk score, as demonstrated by multivariate Cox regression analysis (Figure 7B). The nomogram demonstrated that the risk score is linked to survival rates at 1, 3, and 5 years (Figure 7C). Figure 7D showed satisfactory agreement between observed OS probabilities and OS probabilities predicted by the nomogram, as indicated by the calibration curves for 1-, 3-, and 5-year OS.

### Analysis of tumor immune microenvironment

**Figure 8:**
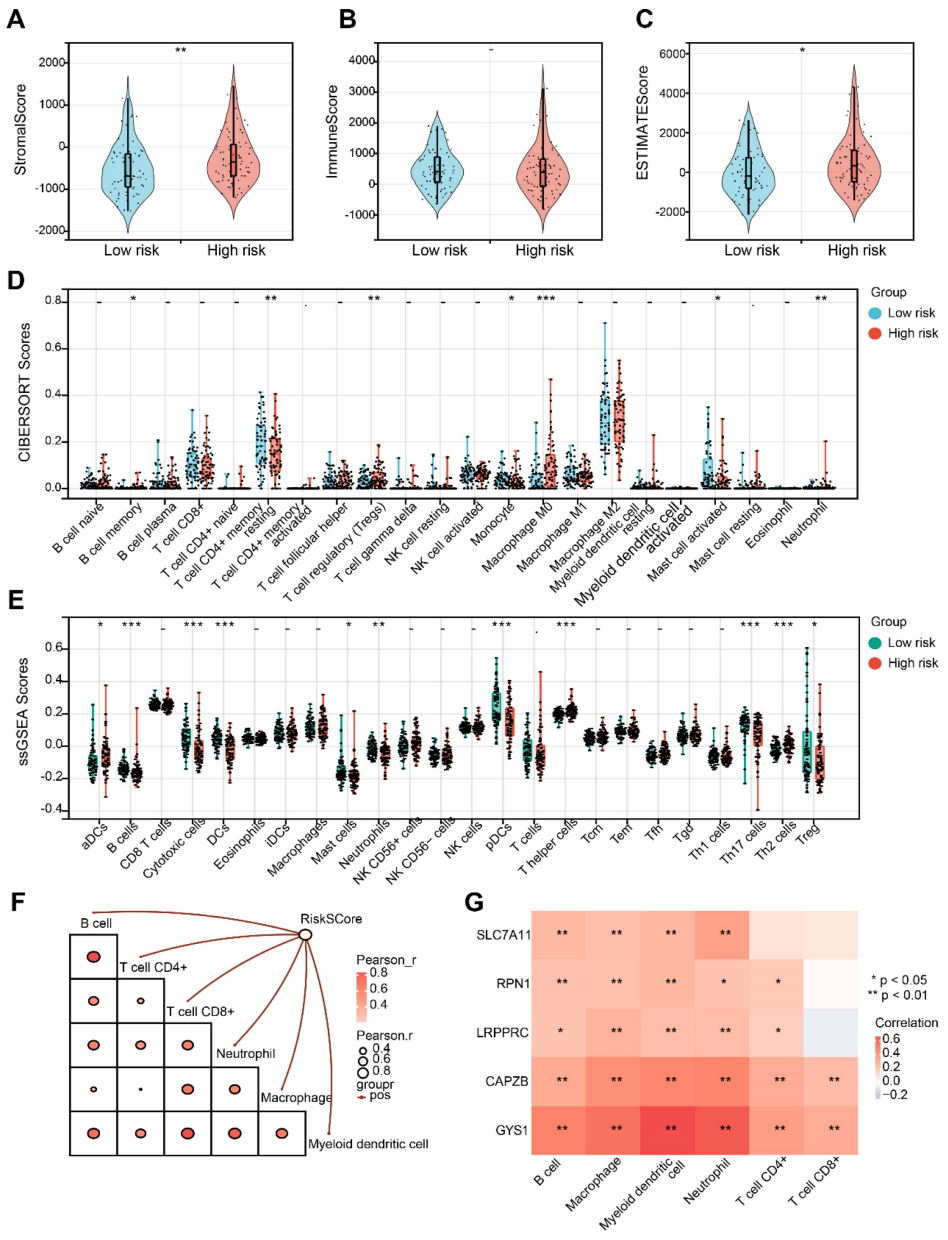
Evaluation of tumor immune microenvironment. (A-C) Distribution of Stromal, Immune and Estimate score in H-group and L-group. (D) Boxplots depicting the CIBERSORT scores of 22 immune cells of the patients in H-group compared to L-group. (E) Boxplots depicting the 29-immune signature ssGSEA scores of the patients in H-group compared to L-group. (F) Correlation of risk score and immune cells based on TIMER. (G) Heatmap describing the correlation between 5 DRGs and immune cells. *P < 0.05, **P < 0.01, and ***P < 0.001.

Initially, it is noticeable that the Stromal score and ESTIMATE score exhibited an increase in the H-group in contrast to the L-group, despite the fact that the Immune score did not demonstrate a significant alteration (Figure 8A-C). Afterwards, CIBERSORT analysis indicated that there were significant differences in B cell memory, resting T cell CD4+ memory, Treg, Monocyte, Macrophage M0, activated Mast cell, and Neutrophil between the L-group and H-group (Figure 8D). Furthermore, utilizing ssGSEA analysis, we observed distinct variations in the infiltration levels of aDCs, B cells, Cytotoxic cells, DCs, Mast cells, Neutrophils, pDCs, Th cells and Tregs in the two groups (Figure 8E). Subsequently, we noticed a substantial positive correlation between the risk score and diverse immune cell populations (Figure 8F). It is important to mention that CAPZB and GYS1 exhibit the highest correlation with the immune cells mentioned above (Figure 8G).

### TMB and MSI analysis

**Figure 9:**
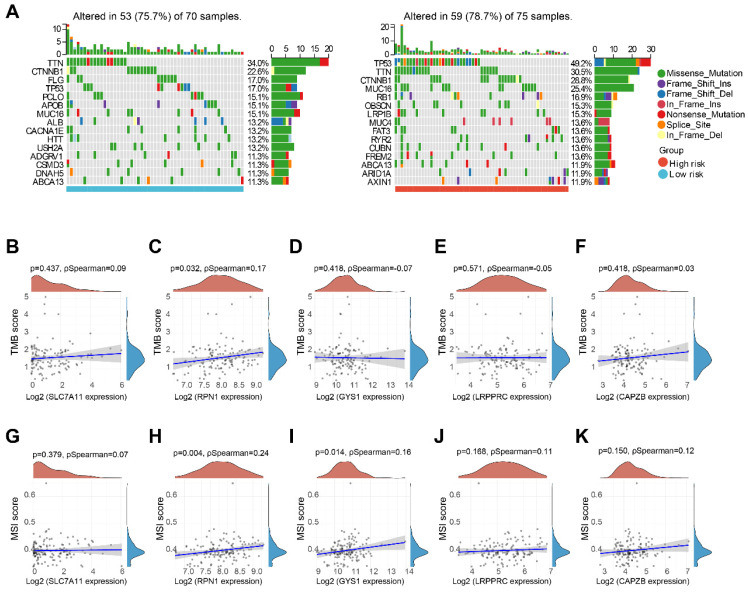
TMB and MSI analysis. (A) Waterfall plot of mutation status in L-group and H-group in HBV-HCC. (B-F) Correlation between 5 DRGs and TMB in HBV-HCC. (G-K) Correlation between 5 DRGs and MSI in HBV-HCC. TMB, tumor mutation burden; MSI, microsatellite instability.

We performed a comparative analysis of somatic tumor mutation profiles in H-group and L-group to explore the correlation between risk scores and TMB. It was observed that that the H-group had a greater occurrence of mutation events (78.7%) compared to the L-group (75.7%). Within the L-group, the most frequently mutated gene was TTN (34%), followed by CTNNB1 (22.6%) and FLG (17%). And in the H-group, TP53 (49.2%), TTN (30.5%), and CTNNB1 (28.8%) emerged as the top three genes with recurrent mutations (Figure 9A). Furthermore, we investigated the correlation between five DRGs and TMB in HBV-HCC, along with MSI. Remarkably, RPN1 exhibited a positive correlation with both TMB and MSI. Additionally, GYS1 demonstrated a significant positive correlation with MSI. However, no significant associations were observed between the other DRGs and TMB or MSI (Figure 9B-K).

### Immune checkpoint and drug sensitivity analysis

**Figure 10:**
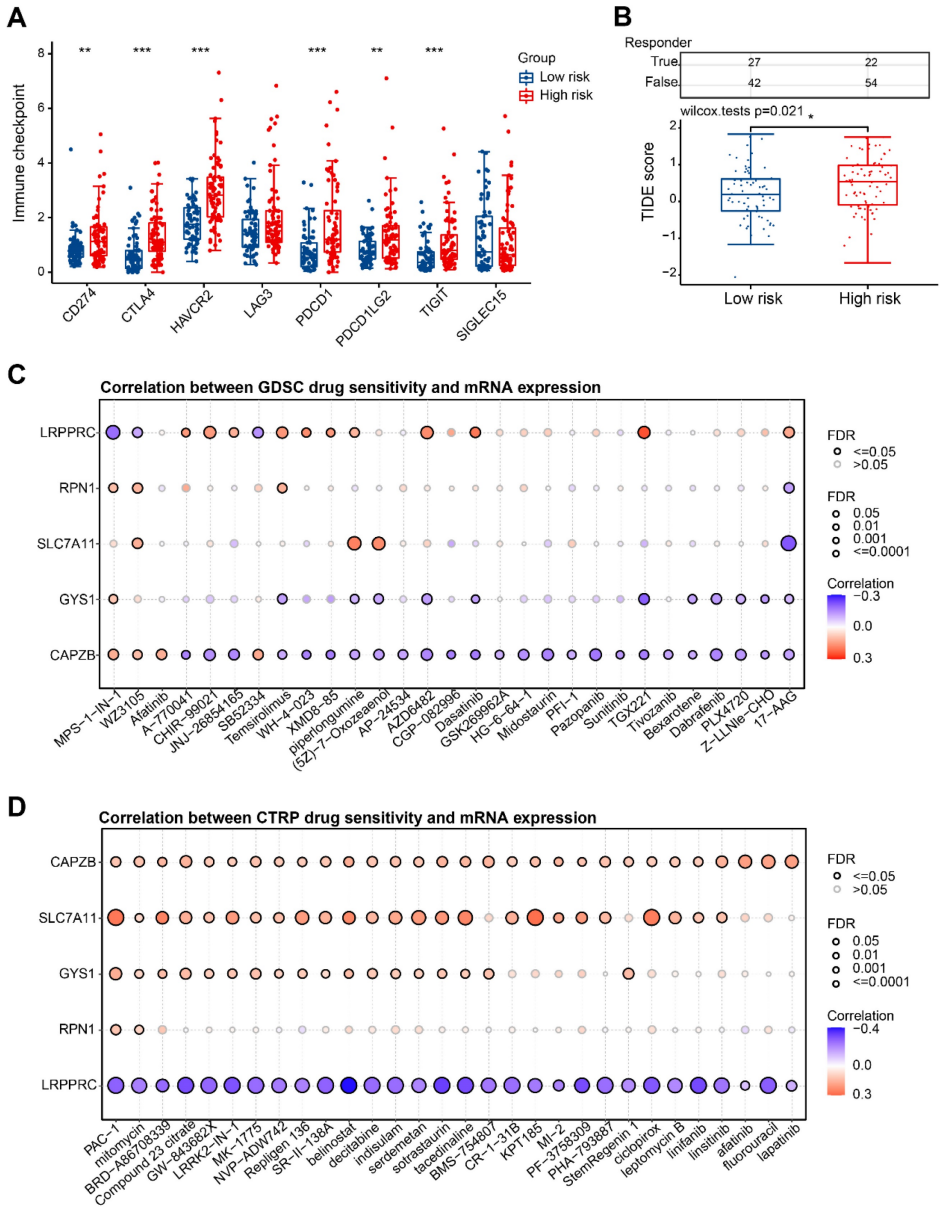
Immune checkpoint and drug sensitivity analysis. (A) Expression levels of immune checkpoints in H-group and L-group. (B) Prediction of TIDE scores for patients in H-group and L-group. (C, D) Correlation of 5 DRGs with various targeted or chemotherapeutic drugs in GDSC and CTRP database. GDSC, Genomics of Drug Sensitivity in Cancer; CTRP, Cancer Therapeutics Response Portal. *P < 0.05, **P < 0.01, and ***P < 0.001.

In our study, we further investigated the potential application of risk score in guiding the clinical treatment of HBV-HCC. Notably, we observed significant up-regulation of immune checkpoint genes in the H-group (Figure 10A). To evaluate the clinical efficacy of immunotherapy in different risk groups, we utilized the TIDE score. The TIDE score is a useful tool that assesses the likelihood of immune evasion, thus indicating the potential benefit of ICB therapy. A higher TIDE score suggests a greater probability of immune evasion, which in turn suggests that the patient may have a lower chance of benefiting from ICB therapy. In our research, we discovered that there was a significantly higher TIDE score in the H-group in comparison to the L-group (P=0.021) (Figure 10B). This implies that patients in H-group may experience compromised effectiveness of immunotherapy due to immune evasion and other related factors. Additionally, we conducted an examination on the interaction between anticancer medications and genes involved in the risk model. It was observed that heightened expression of CAPZB primarily diminishes drug sensitivity according to the GDSC database (Figure 10C). Furthermore, heightened expression of CTRP, SLC7A11, and GYS1 predominantly enhances drug sensitivity as per the CTRP database, while the expression of LRPPRC exhibits a negative correlation with the sensitivity of these drugs (Figure 10D).

### Functional validation of GYS1 in HBV-HCC tumorigenesis and progression *in vitro* and *in vivo*

**Figure 11:**
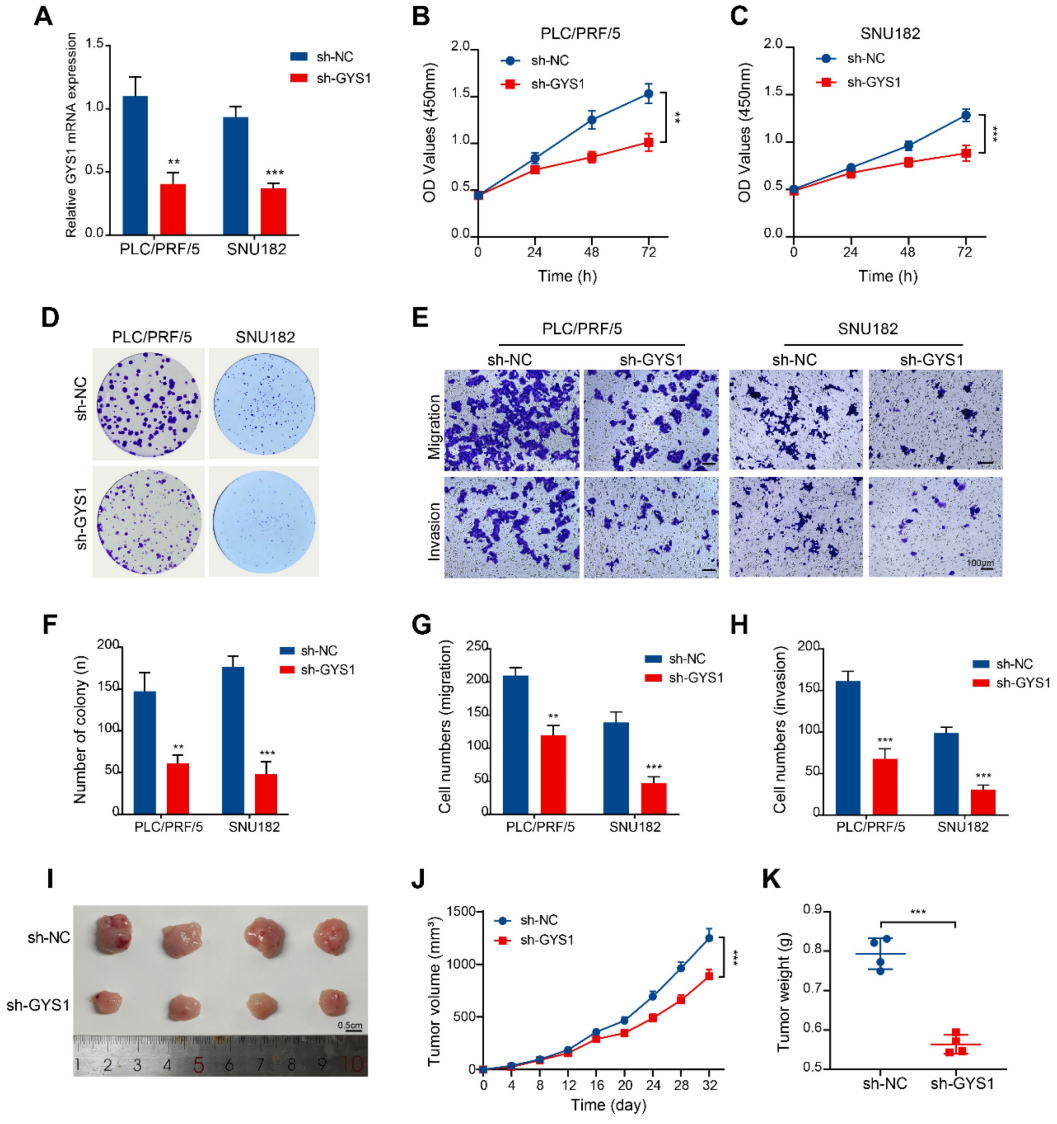
Functional validation of GYS1 in HBV-HCC tumorigenesis and progression *in vitro* and *in vivo.* (A) qRT-PCR detection of GYS1 expression within PLC/PRF/5 and SNU182 cells after transfection with shRNA. (B, C) CCK-8 assays were performed in HBV-HCC cells silenced for GYS1. (D, F) Colony formation of HBV-HCC cells silenced for GYS1. (E, G, H) Transwell migration and transwell invasion assays illustrated that silencing of GYS1 suppress the migration and invasion abilities of HBV-HCC cells. Scale bar = 100 μm. (I) Images of tumor lumps formed by HBV-HCC cells with silenced GYS1. (J, K) Tumor growth curves and weights were analyzed.

The function of GYS1 in HBV-HCC remains uncertain among these 5 prognostic genes. Consequently, our investigation focused on examining the influence that GYS1 exerts on the tumorigenesis and progression of HBV-HCC. We used shRNA to knock down GYS1 in two HBV-HCC cell lines (PLC/PRF/5 and SNU182) and verified the knockdown efficiency by qRT-PCR (Figure 11A). CCK8 and colony formation experiments have demonstrated that suppression of GYS1 led to a notable decrease in both cell growth and the formation of colonies (Figure 11B, C, D, F). Transwell assay was used to evaluate their effects on HBV-HCC cells invasion and migration. We observed a substantial inhibition of cells invasion and migration upon the knock-down of GYS1 (Figure 11E, G, H). To validate the findings of vitro experiments, we established an *in vivo* HBV-HCC model. Our findings demonstrated a notable decrease in both average tumor volume and weight in the sh-GYS1 group in comparison to the sh-NC group (Figure 11I-K). This suggests the crucial role of GYS1 in the progression of HBV-HCC.

## Discussion

MR analysis eliminates potential confounding factors and reverse causality, thereby enhancing the accuracy and reliability of study findings. While HBV infection is the primary catalyst for the development of HCC, there have been no reports investigating the causal relationship between HBV and HCC at the genetic level through MR analysis 22. In this study, we found that increased genetic susceptibility to HBV is a significant risk factor for HCC through MR analysis. Disulfidptosis is a newly discovered form of cell death that is characterized by dysregulated disulfide metabolism and oxidative stress. Wang *et al.* found that disulfidptosis can serve as a valuable diagnostic and prognostic feature of HCC 23. However, the precise causal connection between disulfidptosis and HBV-associated HCC remains uncertain. In this study, the genetic susceptibility to disulfidptosis was identified as an important risk factor for HBV-HCC.

We obtained 21 DRGs from normal and HBV-HCC samples and subsequent functional analyses showed that they were mainly enriched in homotypic cell-cell adhesion, platelet aggregation, regulation of actin cytoskeleton. Interestingly, this study found that some DRGs in the transcriptomic data, such as MYH9, FLNB, DSTN, SLC7A11, ACTB, and FLNA were associated with DNA hypomethylation. Previously, Miao *et al.*'s study found that hypomethylation of glycine dehydrogenase promoter in peripheral blood mononuclear cells is a new diagnostic marker of HBV-HCC 24. In the future, it is necessary to explore the relationship between disulfidptosis -related genes and DNA hypomethylation in HBV-HCC.

Although surgical resection, chemotherapy, and radiotherapy serve as established therapeutic strategies for HBV-HCC, the occurrence of treatment failure, metastasis, and recurrence remains prevalent despite their remarkable efficacy 25,26. Hence, there is a need to undertake risk stratification and prognostic analysis for individuals with HBV-HCC in order to enhance patient outcomes. To accurately predict the prognosis of HBV-HCC, a risk model was developed, consisting of five DRGs. The results of the prognostic prediction using this model were found to be satisfactory. The 5-gene signature used in the model includes GYS1, SLC7A11, LRPPRC, CAPZB and RPN1 as risk factors. Zheng and colleagues' research revealed that the level of RPN1 expression was increased in both HCC cells and tumor samples obtained from patients with HCC. Moreover, it was discovered that circ-SNX27 exerted an impact on the promotion of HCC progression by involving RPN1 27. Numerous investigations have demonstrated that by targeting SLC7A11, it is possible to enhance ferroptosis in HCC cells, thus aiding in the mitigation of HCC's growth and metastasis 28,29. In an m6A-dependent manner, LRPPRC can partially upregulate PD-L1 at the posttranscriptional level, thereby promoting tumor progression and facilitating immune evasion in HCC 30. The results we obtained are generally consistent with these studies. Li and colleagues reported contrasting results whereby the induced overexpression of CAPZB exhibited a notable decline in the migratory and invasive characteristics of HepG2 cells expressing HBxΔ31 31. This suggests that CAPZB may play a protective role in HCC, which is inconsistent with our results. This can be attributed to the limitations of using a simplistic cell model to simulate the complex physiological state of the body. Additionally, it cannot be overlooked that immune cells might also exert significant influence in this context.

Both TMB and MSI are recognized as biomarkers that indicate a positive response to cancer treatment with immune checkpoint blockade therapy 32,33. Using the TMB score, Liu *et al.* developed a risk model that demonstrated the association between elevated TMB score and poorer OS outcomes, as well as increased levels of immune infiltration in HCC 34. The study found that the frequency of gene mutation was significantly higher in the H-group in comparison to the L-group, and the way in which the mutations happened differed. Moreover, a notable correlation was found between the amounts of RPN1 expression and both TMB and MSI. These findings suggest that individuals with heightened RPN1 expression among HBV-HCC patients may display enhanced susceptibility to immunotherapy. The introduction of the TIDE score aimed to establish a more precise indicator of the response to immune checkpoint blockade compared to conventional biomarkers 35. Our observations showed a noticeable elevation in both immune checkpoint levels and TIDE scores within the H-group. These findings strongly suggest that patients categorized as high-risk are in a state of immune suppression. Our data will contribute to the enhancement of DRGs signaling-oriented immunotherapy aimed at enhancing the prognosis of patients with HCC-HBV.

GYS1, a glycosyltransferase family 3 member residing on 19q13, is crucial for the last stage of glycogen synthesis 36. Research has revealed that GYS1 activates the NF-κB pathway, leading to glycogen buildup and fueling the advancement of clear cell renal cancer 37. It should be noted that information regarding the involvement of GYS1 in either HCC or HBV-HCC remains unclear. In our study, it was observed that GYS1 exhibited elevated expression levels among patients diagnosed with HBV-HCC and exhibited a significant correlation with an unfavorable prognosis. By conducting an analysis of the tumor microenvironment, it was determined that the high-risk score group, which included GYS1, displayed a notable increase in both Stromal score and Immune score. Additionally, GYS1 demonstrated a substantial positive association with various immune cell types. Furthermore, the positive correlation between GYS1 and the sensitivity of a vast majority of chemotherapeutic drugs in the CTRP portal indicates that incorporating GYS1-based immunotherapy or chemotherapy into treatment strategies can enhance the likelihood of prognostic improvement for individuals with HBV-HCC. Furthermore, we performed experiments utilizing *in vitro* and *in vivo* models of HBV-HCC to confirm the significance of GYS1. The results from loss-of-function experiments support the notion that GYS1 promotes the growth and spread of HBV-HCC both *in vitro* and *in vivo*. GYS1 is a promising therapeutic target for HBV-HCC based on these findings. However, additional research is necessary to fully grasp the governing mechanisms of GYS1 and its subsequent pathways.

## Conclusion

To summarize, disulfidptosis poses as a potential hazard for HBV-HCC and gene signatures constructed based on DRGs can accurately predict the prognosis of HBV-HCC. GYS1 can promote the progression of HBV-HCC, which is expected to become a new prognostic biomarker and therapeutic target for HBV-HCC.

## Supplementary Material

Supplementary table.

## Abbreviations

AUC: areas under the curve
DRGs: disulfidptosis-related genes
GO: gene ontologyk
HBV-HCC: HBV-associated hepatocellular carcinoma
KEGG: kyoto encyclopedia of genes and genomes
K-M: Kaplan-Meier
LASSO: least absolute shrinkage and selection operator
MSI: microsatellite instability
OS: overall survival
ROC: receiver operating characteristic
TCGA: The Cancer Genome Atlas
TMB: tumor mutation burden
