# Supplementary material for: Unveiling disulfidptosis-related genes in HBV-associated hepatocellular carcinoma: an integrated study incorporating transcriptome and Mendelian randomization analyses
Source: J Cancer. 2024 Aug 26;15(17):5540–56. doi: 10.7150/jca.93194 (PMC11414606; doi:10.7150/jca.93194)
Supplement: Supplementary file 1 — Supplementary table. [file jcav15p5540s1.pdf]

Supplementary Table 1. Clinical characteristics of the HBV-HCC patients used in this study.

	TCGA cohort	GSE14520 cohort
**No. of patients**	145	212
**Age (median, range)**	61 (17-85)	50 (21-77)
**Gender (%)**		
Female	48 (33.1%)	29 (13.7%)
Male	97 (66.9%)	183 (86.3%)
**Grade (%)**		
G1 + G2	99 (68.3%)	NA
G3 + G4	46 (31.7%)	NA
**Stage (%)**		
I/II	90 (62.1%)	165 (77.8%)
III/IV	55 (37.9%)	47 (22.2%)
**T stage (%)**		
T1	46 (31.7%)	NA
T2	44 (30.3%)	NA
T3/T4	55 (37.9%)	NA
**N stage (%)**		
N0	102 (70.3%)	NA
N1/N2	43 (29.7%)	NA
**M stage (%)**		
M0	105 (72.4%)	NA
M1	40 (27.6)	NA
